# Dietary Zinc Limitation Dictates Lifespan and Reproduction Trade‐Offs of *Drosophila* Mothers

**DOI:** 10.1111/acel.14498

**Published:** 2025-01-31

**Authors:** Sweta Sarmah, Hannah Thi‐Hong Hanh Truong, Gawain McColl, Richard Burke, Christen K. Mirth, Matthew D. W. Piper

**Affiliations:** ^1^ School of Biological Sciences Monash University Melbourne Victoria Australia; ^2^ Florey Institute of Neuroscience and Mental Health University of Melbourne Melbourne Victoria Australia

**Keywords:** *Drosophila melanogaster*, lifespan, metal ions, reproduction, zinc

## Abstract

Dietary metal ions significantly influence the lifespan and reproduction of *Drosophila* females. In this study, we show that not adding any of the metal ions to the diet adversely affects reproduction and lifespan. By contrast, food with no added Zn negatively impacts reproduction but does not adversely affect maternal lifespan, indicating it can dictate resource reallocation between key fitness traits. Specifically, it indicates that female flies stop producing eggs to conserve their body Zn for somatic maintenance. Although these data show that flies can sense varying dietary Zn levels to adjust their physiology, they cannot maximise egg production when faced with a choice between food with no added Zn or food with sufficient Zn to support maximum reproduction. Nonetheless, they can choose to preferentially oviposit on Zn‐containing food, perhaps indicating a strategy to assure offspring survival. We also uncovered a role for the *white* gene in sustaining high levels of egg viability when Zn is diluted in the diet. These insights into the role of dietary metal ions, particularly Zn, point to a central role for these dietary micronutrients to indicate environmental quality and so govern trade‐offs between lifespan and reproduction in flies.

## Graphical abstract

Dietary metal ions significantly influence the lifespan and reproduction of *Drosophila* females. In this study, we show that not adding any of the metal ions to the diet adversely affects reproduction and lifespan. By contrast, food with no added Zn negatively impacts reproduction but not maternal lifespan, suggesting that female flies stop producing eggs to conserve their body Zn for somatic maintenance.

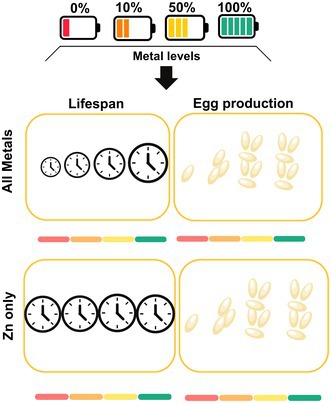

## 1 Introduction

A balanced diet is a central determinant of health, driving changes in growth, reproduction, stress responses, and ageing (Raubenheimer and Simpson 2016; Piper and Bartke 2008). Exactly how dietary nutrient balance is connected to these traits is not fully known, but there is ample evidence to show that excesses or insufficiencies of specific nutrients can be detrimental (e.g., Raubenheimer, Lee, and Simpson 2005; Anderson, Raubenheimer, and Hessen 2020; Simpson and Raubenheimer 2015, 2012). Because nutrient balancing is so important for maximising evolutionary fitness, animals have evolved a wide range of behavioural and physiological adaptations to match nutrient acquisition and metabolism to the available supply (Simpson and Raubenheimer 2012).

Heterotrophic organisms require a mix of both macronutrients (proteins and carbohydrates) and micronutrients (metal ions, vitamins, and sterols) in their diets (Sang 1961; Piper et al. 2014; Mirth, Nogueira Alves, and Piper 2019). Unlike macronutrients, which are required in relatively large amounts to build biological structures and for use as a source of energy, micronutrients are required in smaller amounts and are mostly required to facilitate metabolic processes (e.g., as enzyme co‐factors) and to maintain the physical environment (Bhattacharya, Misra, and Hussain 2016). Because of their relatively greater abundance and their role in energy generation, most research on the eco‐evolutionary significance of dietary nutrition has focused on macronutrients, with relatively less emphasis on micronutrients.

In *Drosophila melanogaster* and other insects, several studies have shown that increasing the ratio of dietary protein relative to carbohydrate can promote adult reproduction, but at the cost of reduced lifespan, while diets with lower protein‐to‐carbohydrate proportions reduce reproduction and increase lifespan (Mair, Piper, and Partridge 2005; Piper and Partridge 2011; Simpson et al. 2017; Lee et al. 2008; Skorupa et al. 2008). More recent data has shown that the reciprocal relationship between these traits is conditional on the quality of protein provided and the levels of sterol in the food: For female *D. melanogaster* , a combination of small amounts of high‐quality protein with ample amounts of the micronutrient cholesterol provides sustenance for both long lifespan and high reproduction simultaneously (Piper 2017; Zanco et al. 2021). These data show that micronutrients can mediate the way that macronutrients affect fitness.

We have also found that manipulating the levels of some metal ions, which are also essential micronutrients for development, plays a central role in determining the fitness of female flies by controlling rates of reproduction and their relationship to lifespan (Piper et al. 2014). These data indicate that flies sense the levels of one or more metal ions in the process of determining the degree to which they commit resources to reproduction versus adult survival. Despite this apparent central role in life history trait trade‐offs, the scope of fitness responses to dietary metal ions remains understudied.

Metal ions are important structural and catalytic components that support a wide range of processes that collectively contribute to an organism's overall well‐being. For example, iron (Fe), copper (Cu), magnesium (Mg), manganese (Mn), and zinc (Zn) all function as catalysts in various enzymes involved in energy generation, amino acid metabolism, and cellular redox metabolism (Jomova et al. 2022; Dow 2017). Additionally, Zn is integral to the structural integrity and function of proteins engaged in DNA repair, transcriptional regulation, and translational processes, while iron is involved in oxidative stress mitigation, and copper is a vital cofactor for enzymes participating in respiration and pigmentation (Vilas et al. 2018; Galaris, Barbouti, and Pantopoulos 2019; Tsang, Davis, and Brady 2021). These molecular functions underpin important physiological processes, and the absence of these metal ions in the diet has been linked to growth inhibition, sterility, loss of circadian rhythms, and immunodeficiency (Hu et al. 2020; Sang 1961; Tian et al. 2014; Iatsenko et al. 2020; Rudisill et al. 2019; Kim et al. 2010; Tian and Diaz 2012; Que et al. 2015; Horner and Wolfner 2008; Pankau and Cooper 2022).

Zn plays a crucial role in many biological processes, including egg activation in *Drosophila* and other organisms (Hu et al. 2020). Maintaining proper Zn homeostasis is essential for normal development and reproduction. In *Drosophila*, Zn is stored in specialised storage granules in the Malpighian tubules, which are analogous to the kidneys in mammals (Navarro and Schneuwly 2017). The formation and maintenance of these Zn storage granules depends on Zn transporters that regulate the flow of Zn into and out of cells and organelles. One important gene involved in Zn homeostasis in *Drosophila* is the *white* gene. *White* encodes a protein that functions as a pigment transporter required for normal eye colour, but recent studies have revealed it also plays a key role in whole‐body Zn homeostasis (Tejeda‐Guzmán et al. 2018). The White protein is involved in sequestering Zn in storage granules in the Malpighian tubules (Mackenzie et al. 1999; Yin, Qin, and Zhou 2017; Tejeda‐Guzmán et al. 2018). We have extended these findings by examining how mutations in the *white* gene alter egg production, viability, and maternal lifespan under conditions of dietary Zn limitation.

Here, we explore the effects of dietary metals on adult female egg production, behaviour, and lifespan in *D. melanogaster* . Interestingly, with the addition of no metal ions to the food, we observe a general reduction in reproduction and lifespan, but manipulating only dietary Zn results in a lifespan/reproduction trade‐off similar to that observed when dietary protein:carbohydrate ratios are altered. These data indicate that flies have physiological sensing mechanisms that enable them to measure dietary Zn supply and adjust their life history strategies accordingly. These observations offer important insights into how dietary components, in particular the micronutrients, shape evolutionary fitness.

## 2 Results

### 2.1 Dietary Metal Ions Are Required for Lifespan and Reproduction in *Drosophila* Females

To examine the effects of dietary metal ions on *Drosophila* lifespan and fecundity, we used completely defined, synthetic (holidic) diets (Piper et al. 2014; Piper 2017). Four different experimental diets were created by diluting the mixture of metal ions (Ca, Cu, Fe, Mg, Mn, and Zn) to 0%, 10%, 50% and 100% of the level in the complete (control) diet, while all other components were identical between foods (Figure S1A). We tested fecundity and lifespan responses to these treatments using two genetically matched outbred strains of *Drosophila*, one wild‐type (red‐eyed Dahomey; *rDah*) and the other lacking the *white* gene (white‐eyed Dahomey; *wDah*).

**FIGURE 1:**
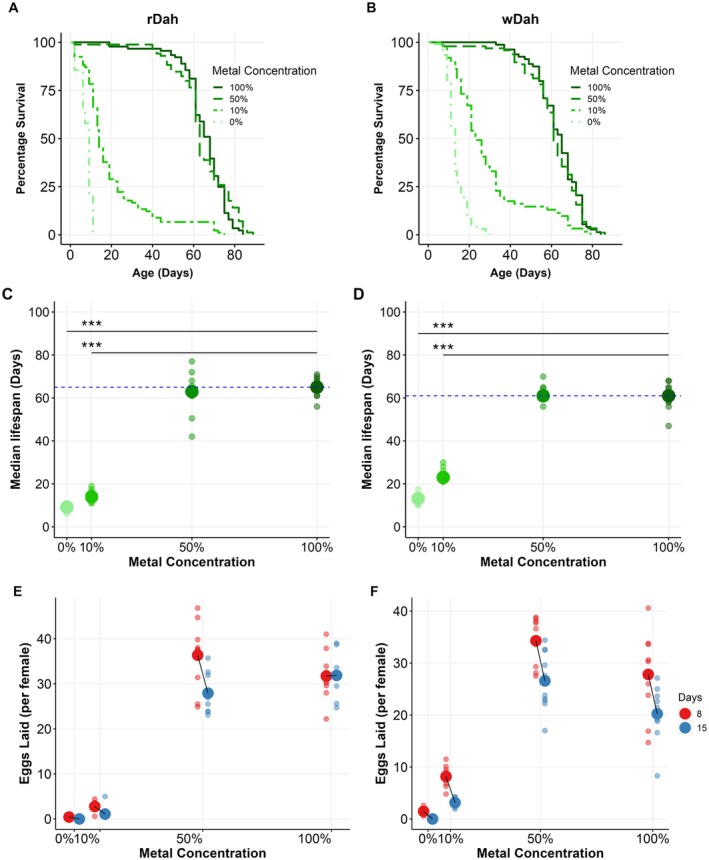
Diluting dietary metal ions reduces the lifespan and fecundity of *Drosophila*. (A) rDah and (B) wDah females demonstrate a significantly reduced lifespan with metal ion dilution below 50% of the level in control food. (C) and (D) Replicate (small circle) and median (large circle) lifespans for data illustrated in panels (A) and (B), respectively. The horizontal dashed blue line denotes the median lifespan of the ‘100%’ metal ion (fully fed control) group. The asterisks indicate significant differences from 100% metal ions (Table S2). (E) rDah females and (F) wDah females display a similar, but statistically different (Table S3), decline in egg laying over time and in response to metal ion dilution. Asterisks indicate significant lifespan differences from the 100% metal ion control group (***p < 0.001; see Table S2 for details).

Both *rDah* and *wDah* suffered progressively shortened lifespans as metal ions were diluted below 50% of the level on control food to 10% and 0%, but they did so to significantly different extents (Figure 1A,B, Table S1). In particular, *rDah* flies showed a greater lifespan shortening at 10% and 0% metal ions (medians: ~14 and ~9 days respectively) than *wDah* (medians: ~23 and ~13 days respectively; Figure 1C,D, Table S2).

Fecundity, and its decline with fly age, were also modified by metal ion dilution, and these effects were also altered by the flies' genotype (Figure 1E,F, Table S3). Specifically, the egg production of flies fed 0% and 10% metal ions was significantly reduced from that of flies on 100% control food, and this reduction was more pronounced in *rDah* than *wDah* flies. These findings demonstrate the essential nature of dietary metal ions to *Drosophila* adult females for egg laying and lifespan. They also show that the *white* transporter has a role to play in egg production and lifespan during metal ion limitation.

### 2.2 Genotype Modifies the Lifespan and Fecundity Responses to Individual Metal Ion Restrictions

**FIGURE 2:**
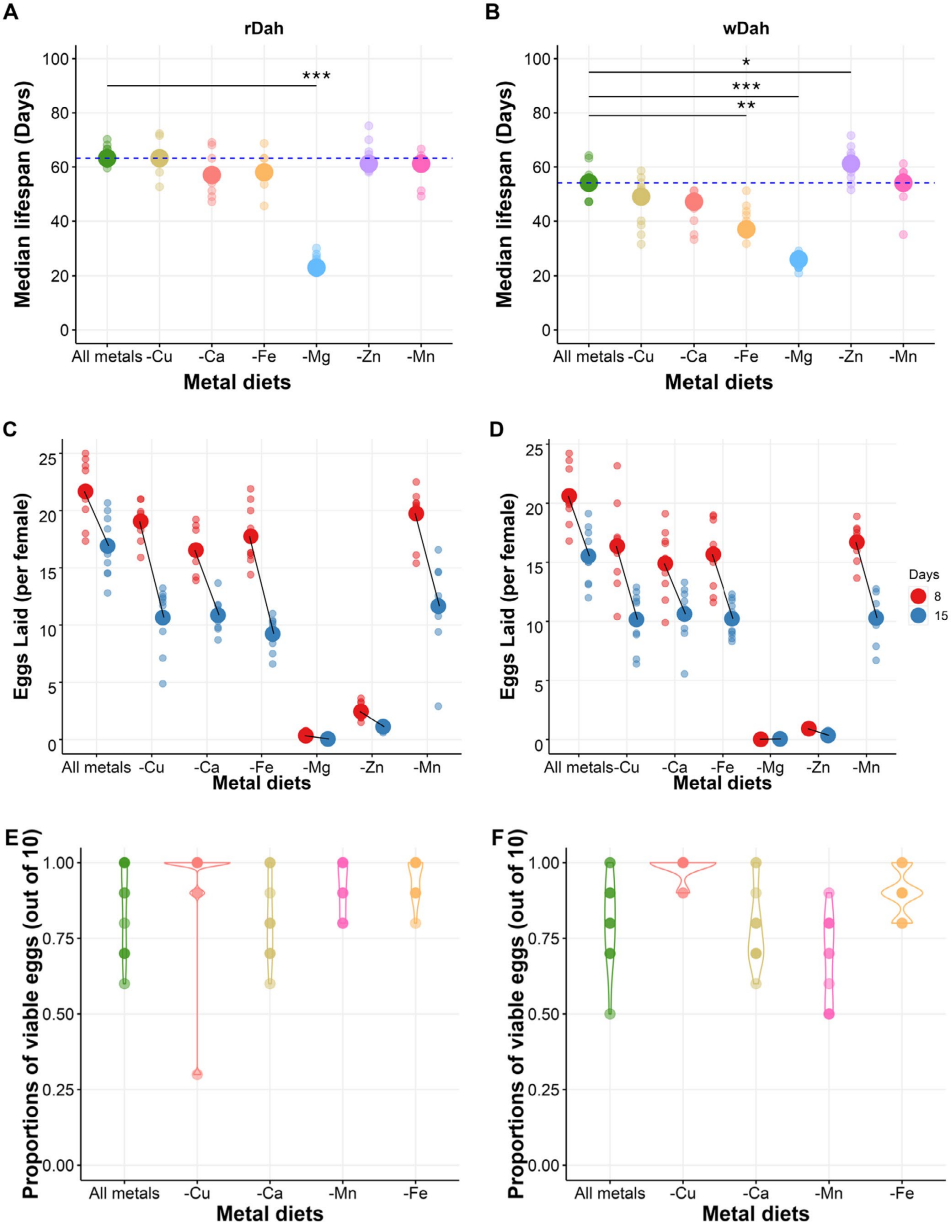
Individual metal ions not being added elicit distinct outcomes for both lifespan and reproduction. For rDah flies (A), not adding magnesium resulted in significant lifespan shortening. In contrast, wDah flies (B) exhibited a significant lifespan cost when Fe or Mg were not added and a small but significant lifespan benefit when Zn was not added. Replicate means are represented by small circles, while larger circles represent population median. To facilitate comparisons between treatment groups, a dashed horizontal line marks the mean lifespan of the ‘All metal’ treatment group. The asterisks indicate a significant difference from “All metals”. Full lifespan curves are shown in Figure S2. After exposure to each diet for 8 or 15 days of adulthood, egg laying was relatively unaffected, except for when flies were exposed to foods where Zn or Mg were not added, on which both rDah (C) and wDah (D) showed strongly reduced egg production. For both rDah (E) and wDah (F), egg‐to‐adult viability was not compromised across different individual metal ions not being added (Ca, Cu, Fe, and Mn). Asterisks indicate significant lifespan differences from the “All metals” control group (***p < 0.001; **p < 0.01; *p < 0.05; see Table S5 for details).

Since metal ions are essential for egg production and adult lifespan, we wondered if the flies suffered equally detrimental outcomes from deprivation of any one metal ion or if these responses would be distinct, which could indicate differences in their biological and ecological significance. To answer this question, we restricted each of the metal ions individually in the diet and measured fly lifespan and fecundity (Figure 2, Figure S2).

The effects of individual metal ion restrictions on lifespan differed according to the identity of the metal ion excluded, and this effect was modified by genotype (Figure 2A,B, Figure S2B,C, Table S4). Specifically, there was very little effect of each individual metal ion restriction on *rDah* lifespan, except for when Mg was not added, which shortened median lifespan from ~62 to ~23 days. By contrast, the responses of *wDah* varied more, as not adding Fe, Mg, and Zn all significantly modified lifespan to some extent (Figure 2B, Figure S2C). Specifically, *wDah* median lifespan was shortened when Fe and Mg were not added, while surprisingly, not adding Zn caused a mild but significant extension to median lifespan (Figure 2B, Figure S2C, Table S5).

Individual metal ions not being added to the diet also elicited distinct outcomes for fecundity, but these effects were not modified by genotype (Figure 2C,D, Table S6). The most striking dietary effects were caused by not adding either Mg or Zn to the food, which resulted in an arrest of egg production by day 8 for both genotypes (Figure 2C,D, Table S7). We also observed a small, but significant, reduction in egg laying on day 8 when either Ca or Fe was not added, while not adding Cu or Mn did not differ significantly from the fully fed controls (all metals). However, on day 15, non‐addition of any one of the metal ions (Ca, Cu, Fe, and Mn) showed significantly reduced egg production when compared to the fully fed controls for both genotypes (Figure 2C,D, Table S7).

When adult female flies encounter food with inadequate levels of Ca, Cu, Fe, or Mn, continuing to lay eggs at a high rate early in life may be an effective strategy to maximise overall fitness as long as the eggs laid are still adequately provisioned with nutrients to be viable. To assess this, we collected eggs from females maintained on diets where each of these single ions was not added for 8 days and then transferred the eggs to a yeast‐based diet (Bass et al. 2007), which is nutritionally complete, to support larval development. For both *rDah* and *wDah*, restriction of any one of the metal ions did not modify the proportion of eggs that produced viable adults (Figure 2E,F, Table S8). Thus, although lacking any one of these metal ions from the adult diet can be costly for maternal lifespan, early fecundity and offspring production remain unaffected by limiting amounts of any one of these nutrients.

Taken together, these data demonstrate that female flies implement at least three different strategies when encountering restriction of an essential metal ion: (1) they continue to produce and lay viable eggs with either no or only a relatively small detrimental effect on maternal lifespan (observed for Cu, Ca, Fe, or Mn restriction); (2) they cease egg production and preserve maternal lifespan (observed for Zn restriction); or; (3) they cease producing eggs and die young (observed for Mg restriction).

### 2.3 Zn Dilution Reduces Reproduction, but Maternal Lifespan Is Not Compromised

**FIGURE 3:**
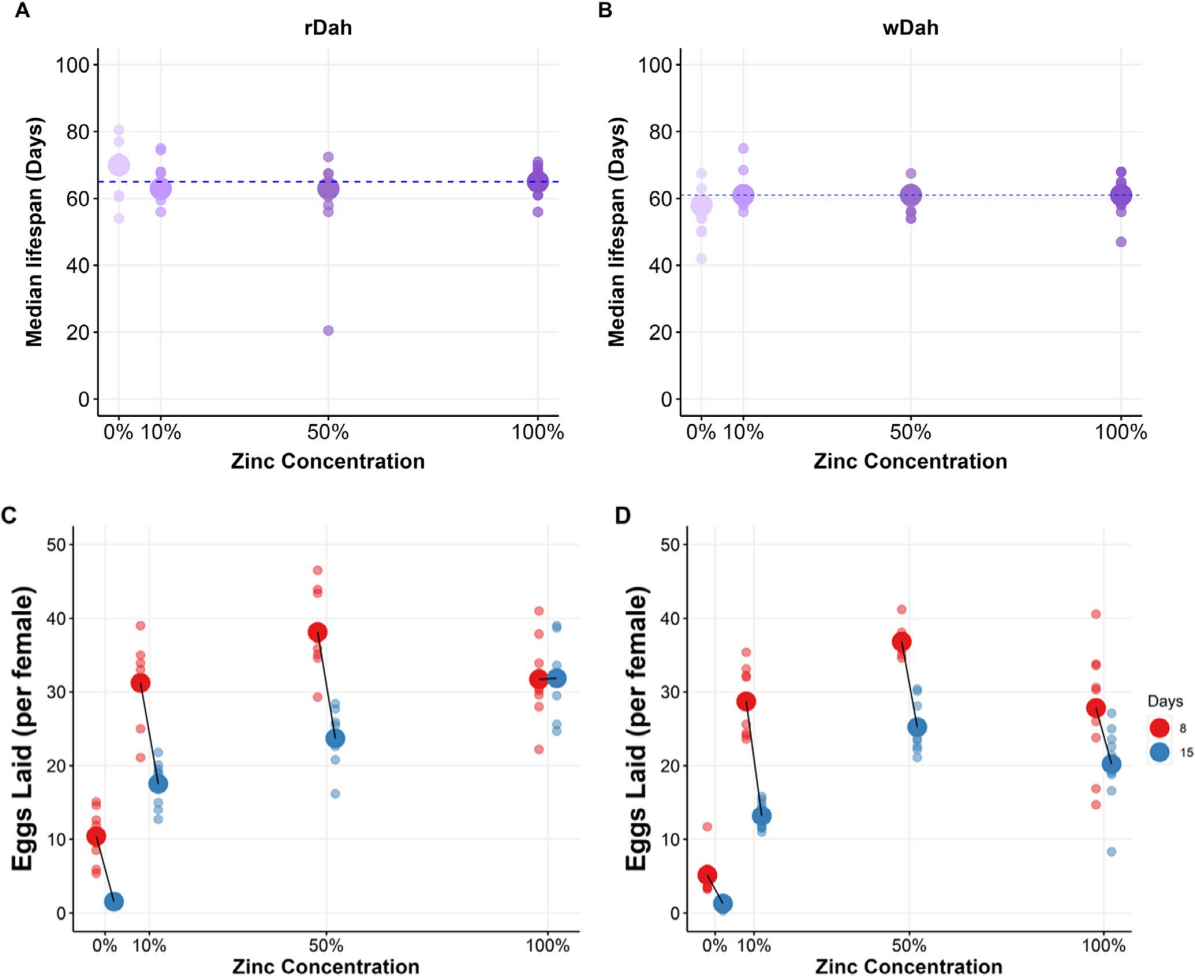
Dietary Zn dilution reduces reproduction with a small increase in lifespan. Dietary Zn dilution had little effect on rDah (A) and wDah (B) lifespan. Population medians are shown as larger circles, and smaller circles represent replicates. The dashed horizontal line at the median lifespan value represents the ‘100%’ Zn control group. Full lifespan curves are shown in Figure S3. Both rDah (C) and wDah (D) females display a significant decline in egg laying over time and in response to Zn dilution. The two genotypes differed significantly in the dynamics of their responses to Zn dilution (Table S11).

Not adding Zn to the diet produced a different outcome compared to when any of the other metal ions were restricted: the flies rapidly ceased egg production while preserving maternal lifespan. To explore this further, we created four different experimental diets containing Zn at 0%, 10%, 50% and 100% of the level in the complete (control) diet (Figure 3, Figure S3).

There was no reduction in lifespan for either *rDah* or *wDah* flies at any level of Zn dilution (Figure 3A,B, Figure S3B,C, Tables S9 and S10). Interestingly, we did not observe an extension of median lifespan in this experiment, indicating that the benefit of Zn restriction in the previous lifespan experiment is small and variable.

Reducing Zn in the diet caused a graded reduction in egg laying that was similar for both genotypes, although the exact pattern of change was significantly modified by genotype (Figure 3C,D, Table S11). Notably, both *wDah* and *rDah* flies exhibited only a small reduction in egg laying with 10% Zn, but a strong reduction with 0% Zn (Figure 3C,D).

One explanation for why flies on Zn‐restricted diets shut down egg production and maintain adult lifespan is that they simply eat less food. If true, the flies would be eating less protein, meaning that macronutrient restriction, rather than Zn restriction, might account for the effects we have observed (Mair, Piper, and Partridge 2005; Lee et al. 2008; Skorupa et al. 2008). To assess this, we measured the change in metal ion levels in flies feeding on food to which Zn was not added.

**FIGURE 4:**
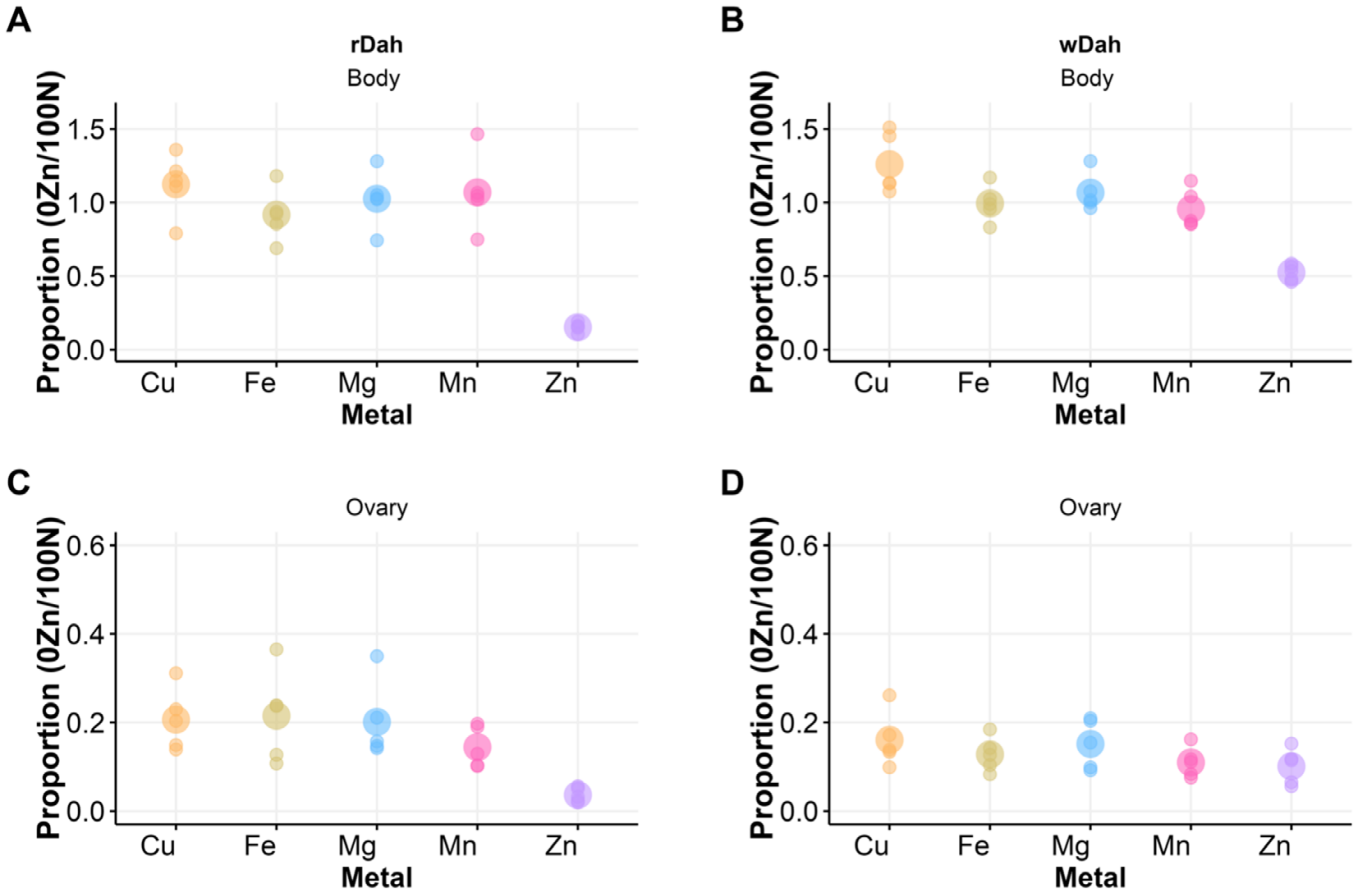
Genotype and diet influence metal ion levels in the ovary and body of flies. Proportions of metal ions in the bodies of (A) rDah and (B) wDah flies were significantly modified by dietary Zn levels. Specifically, Zn levels were strongly reduced in the bodies of Zn‐restricted flies. In the ovary, metal proportions (Cu, Fe, Mg, Mn, Zn) were significantly reduced in flies on Zn‐restricted diets in both (C) rDah and (D) wDah, perhaps reflecting a reduction in ovary size as Zn restriction suppresses egg production.

We separated ovaries from the remaining body tissue of *rDah* and *wDah* flies and measured the levels of Zn, Cu, Fe, Mg, and Mn using Inductively Coupled Plasma Mass Spectrometry, comparing the proportions of each metal in flies on a 0% Zn diet with those on a complete diet (100N; Figure 4). Metal ion deprivation was evident in both tissue types in both genotypes but occurred to different extents for each metal (Table S12). After 8 days of feeding on food with no added Zn, we observed a specific reduction in Zn in the body samples when compared with fully fed flies (Figure 4A,B). This effect was more pronounced in *rDah* (~5‐fold lower) than *wDah* (~2.5‐fold lower) (Table S13). All other metal ions in the bodies of flies feeding on food where Zn was not added were either unchanged or, in the case of Cu, exhibited a small but significant increase in abundance (Figure 4A,B, Table S13). This pattern of change indicates that the flies are maintaining the intake of all nutrients, other than Zn, for which they are specifically experiencing a reduction, which is consistent with the flies not reducing overall food intake on diets where Zn was not added.

In the ovary samples, Zn was also drastically reduced by at least 10‐fold when compared to fully fed flies (Figure 4C,D, Table S13). In this case, however, all other metal ions measured also showed ~5‐fold lower levels than what was found in fully fed animals. Given that these flies still retained normal levels of the metal ions in their body tissue (Figure 4A,B), we assume that this is not specific depletion of all metals from the ovaries but instead reflects a generalised reduction in ovary size that accompanied cessation of egg production (Kosakamoto et al. 2024). Interestingly, although both sets of fly tissues show substantial Zn loss when feeding on Zn‐restricted diets, the flies still retain enough Zn to sustain vital functions that support full lifespan.

### 2.4 Females Cannot Maximise Egg Production When Offered a Choice of Diets Varying in Zn Levels, but They Do Exhibit Oviposition Site Preference

Our data show that flies on Zn‐restricted diets cease egg production and lose a substantial proportion of their body Zn levels. We therefore decided to test if the flies could modify their behaviour and/or physiology to maximise fitness when a food choice is available that would allow them to avoid Zn restriction. To do this, we gave groups of females one of several pairwise food choices, in which only the Zn concentration varied, and counted the total number of eggs laid.

**FIGURE 5:**
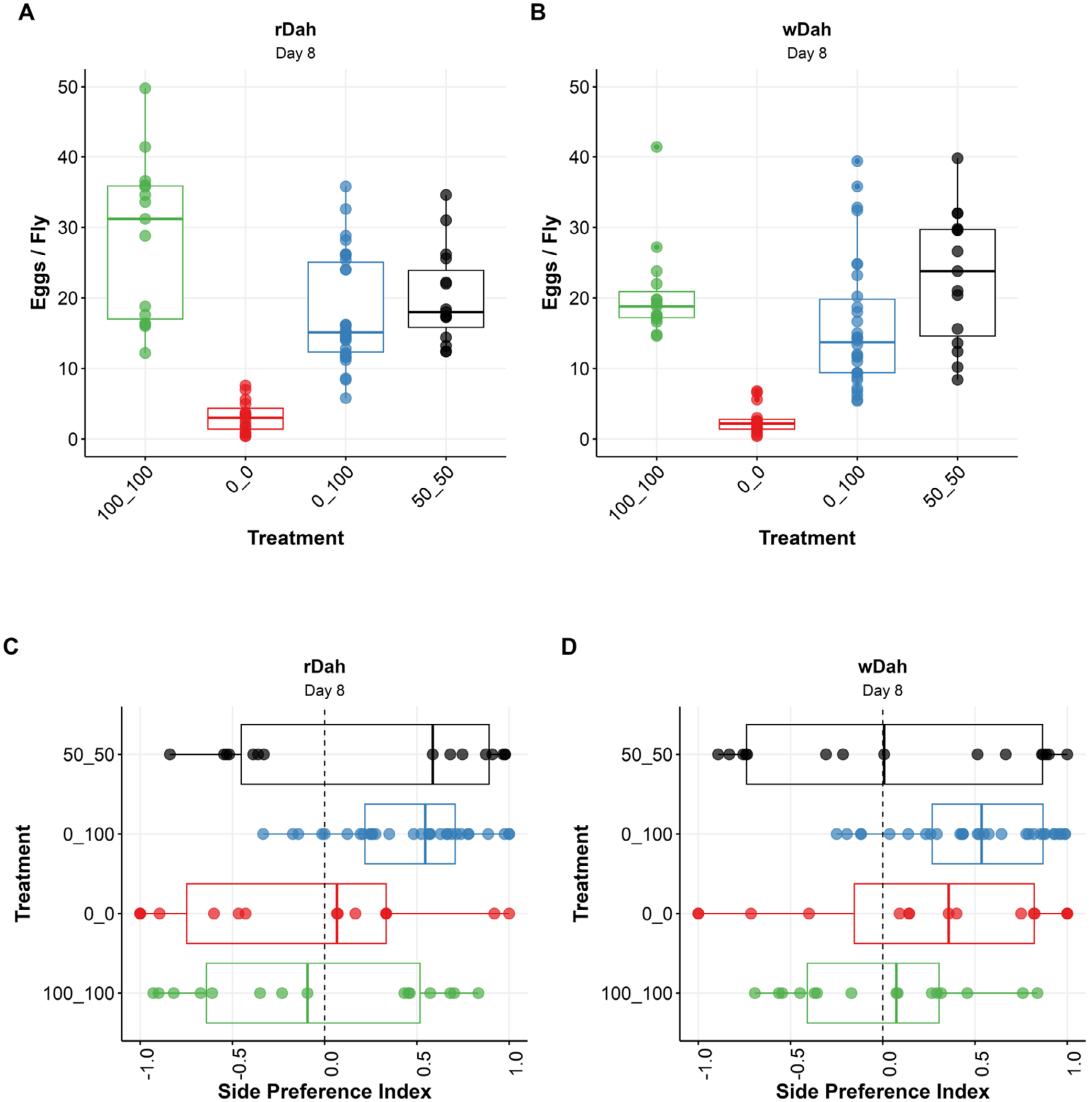
Given pairwise choices of food varying only in Zn content, flies do not optimise egg production but do select Zn‐containing food for oviposition. After 8 days of exposure to a choice of two diets that differed only in Zn concentration, rDah flies (A) and wDah flies (B) laid eggs at a rate that reflected random feeding between the two foods. (C) rDah and (D) wDah flies on day 8 showed a preference to oviposit on food containing Zn over food lacking Zn. This phenotype appeared to strengthen as the assay duration increased (see Figure S5 for day 2 and day 15 data for both assays) (Tables S16 and S17).

The diet pairs we used were a positive control in which both food options contained 100% Zn (100_100); a negative control in which both foods contained 0% Zn (0_0); and an experimental condition in which the flies could choose between one food containing 0% Zn and another containing 100% Zn (0_100). To control for the situation where flies randomly sampled the two food options in the experimental condition and so effectively consumed 50% Zn, we added a third control, in which both food options contained 50% Zn (50_50) (Figure S4). For all conditions, we counted eggs laid in a 24‐h window after continuous exposure to the diet pairs for 2 days (Figure S5A,B), 8 days (Figure 5A,B), and 15 days (Figure S5C,D).

For both genotypes, egg production changed in response to the diet choice conditions and the age at which egg laying was assessed (Figure 5A,B, Figure S5A–D, Table S14). At the earliest time point (day 2), egg‐laying was mostly indistinguishable across all dietary pairs, indicating that Zn‐mediated changes in egg production occur more than 2 days after being introduced to the new foods (Figure S5A,B, Table S14).

By day 8 of adulthood, egg production had diverged between diet choice groups (Figure 5A,B, Tables S14 and S15). For both genotypes, egg production was almost completely arrested for flies without Zn (0_0). For *rDah* flies, egg laying significantly increased when flies were maintained in the 50_50 choice and increased further again for flies confined to the 100_100 choice. These data indicate that egg laying was Zn limited and thus egg production reflected diet composition. When given the experimental diet pair (100_0 choice), egg numbers were higher than for flies in the 0_0 condition, lower than for flies on the 100_100 condition, and no different from the flies that were maintained on the 50_50 diet pair (Figure 5A, Table S15). These data show that when flies are given a choice between a diet that cannot sustain any egg production (0% Zn) and another that sustains maximum egg production (100% Zn), they do not alter their behaviour and/or physiology to maximise egg production. Although we did not measure food consumption, this is consistent with a situation in which the flies cannot distinguish between food containing 0% Zn and that containing 100% Zn and so consume food randomly from both diets, which limits egg production to the same level as when they only have food with 50% Zn available.

At this same time point (day 8), *wDah* flies in the choice situation (0_100) also produced more eggs than the flies in the 0_0 condition (Figure 5B). However, unlike *rDah*, the flies with the 0_100 choice produced eggs at an equally high rate as flies on 100_100 (Figure 5B, Table S15). Surprisingly, *wDah* flies maintained on the 50_50 control diet pair also had higher than expected egg production, such that it was also indistinguishable from the positive control (100_100) (Figure 5B, Table S15). This indicates that dropping Zn to 50% of the level of that in the full feeding condition did not limit egg production of *wDah* females like it did for *rDah* females.

When assaying the flies on day 15, both genotypes showed the same egg‐laying trends across food types as what they showed on day 8, but most differences between diet choice groups were reduced as egg laying dropped due to natural age‐related decline in egg production (Figure S5C,D, Table S15).

Together, these findings suggest that while dietary Zn levels can be physiologically limiting for egg production, the flies showed no evidence for an ability to counteract the negative effects of Zn limitation on egg production when given a choice of foods in which the level of Zn alone varies.

Because of the way our assay is set up, we could also assess whether mothers used the presence of dietary Zn as a criterion for selecting the food on which they lay their eggs. When considering all our diet pairs, we found that the flies laid more eggs on Zn containing food than food lacking Zn (Figure 5C,D, Figure S5E–H, Tables S16 and S17). Although the strength of this egg site selection differed between genotypes, both demonstrated a stronger preference for Zn‐containing food the longer the assay continued. This analysis indicates that the flies express a diet‐based choice for egg‐laying site selection that is sensitive to dietary Zn levels. However, it does not rule out the influence of other cryptic preferences that the flies may be expressing, which could produce the same outcome by chance. Examples include the tendency for conspecifics to lay eggs at the same site as a leading female (Sarin and Dukas 2009; Moreira‐Soto et al. 2024) or some visible cues in the experimental setup that we are unaware of.

To test if the site selection was indeed specific to dietary Zn levels, we assessed if the flies' laying site choices in the 0_100 condition significantly differed from egg‐laying site preferences demonstrated by flies with pairs of foods that were nutritionally identical. For both genotypes, there was little evidence that egg site selection for the flies in the 0_100 choice differed from egg‐laying site selection bias shown in controls after only 2 days of exposure to the choice (Figure S5E,F, Tables S16 and S17). However, the strength of site selection in the 0_100 condition increased beyond any site bias in at least two of the three controls by day 8 (Figure 5C,D, Table S17) and in both controls by day 15 (Figure S5G,H, Table S17). Thus, both genotypes exhibited a growing strength of preference to lay eggs on food containing Zn over food with no added Zn over time. Given that maternal survival and feeding behaviour appear to be unaffected by dietary Zn restriction, this egg site preference may indicate that when maternal Zn stores drop, they select an egg‐laying site that protects larval survival, which relies on dietary Zn (Consuegra et al. 2020).

### 2.5 The *White* Gene Is Required to Maintain Egg Quality Control During Dietary Zn Limitation

**FIGURE 6:**
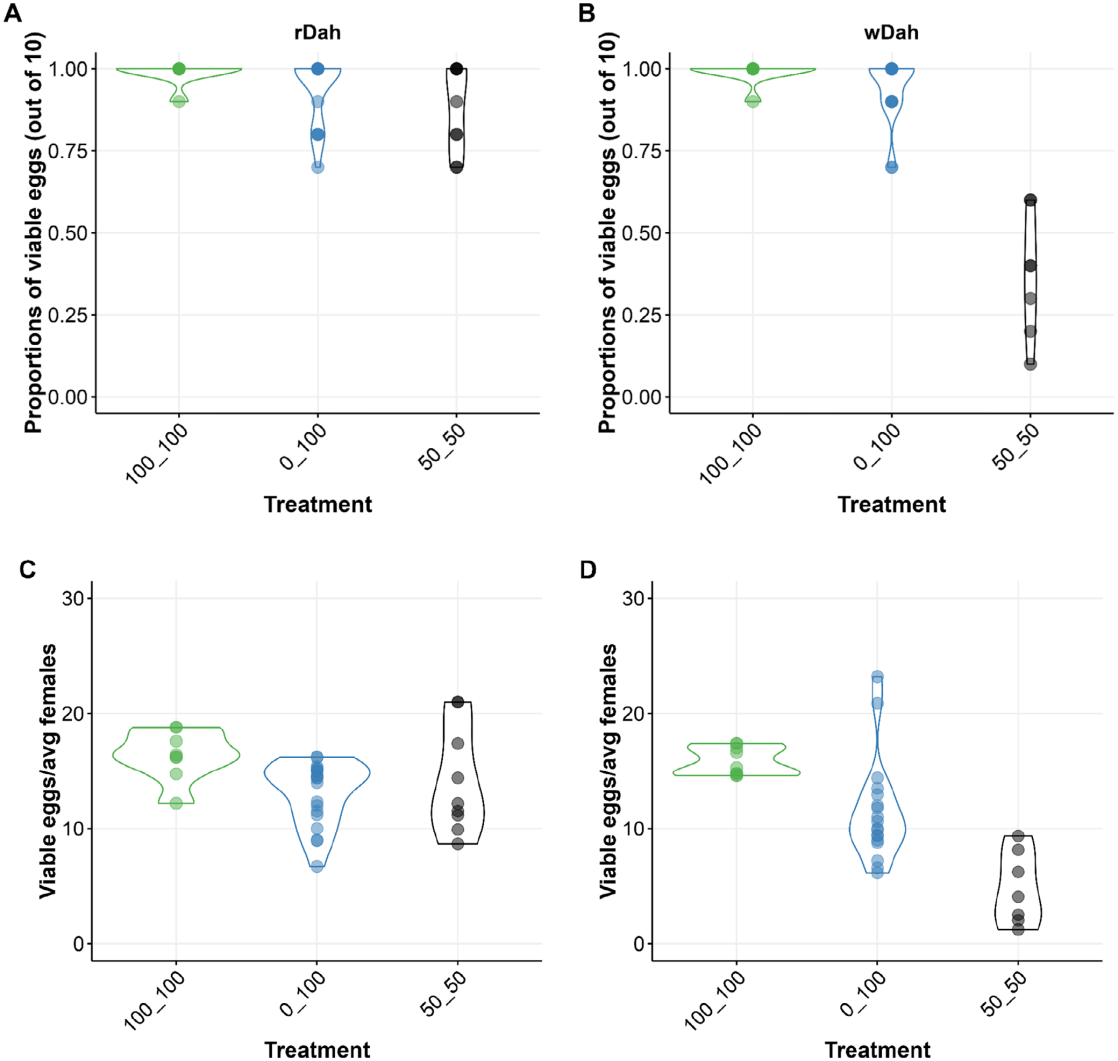
Egg viability is compromised when wDah, but not rDah, flies feed on Zn‐limiting diets. The proportion of viable eggs laid by (A) rDah mothers remains unchanged and high for flies that fed on any of the diet pairs tested in which Zn was manipulated (100_100, 0_100 or 50_50). By contrast, the proportion of viable eggs produced by (B) wDah mothers was significantly reduced by Zn limitation, which meant that, unlike (C) rDah mothers, (D) wDah females laid overall fewer viable eggs when feeding on Zn‐limiting diets.

All of our data above show that the mutation in the *white* gene modifies egg‐laying and lifespan responses of flies to dietary Zn levels, albeit in relatively minor ways. One of the stronger phenotypes was the combined observation that in the choice assay, *wDah* females on food with 50% Zn laid eggs at the same rate as when on 100% Zn (Figure 5B), but they also retained a relatively high proportion of Zn in their body tissue when feeding on food with no added Zn (Figure 4B). We, therefore, wondered if *wDah* females had dysregulated egg production, such that they laid more but lower‐quality, eggs when Zn was limited. To assess this, we measured the egg‐to‐adult viability of a sample of eggs collected on day 8 from all diet pairs used in the diet choice assay above (except 0_0, which produced insufficient eggs for the assay) (Figure 6).

Genotype significantly modified the proportion of viable eggs laid by females across the different diets (Figure 6A,B, Table S18). Specifically, while egg viability was unchanged and fixed at > 80% for both genotypes maintained on most diet pairs, when *wDah* flies fed on the 50_50 diet, egg viability was significantly lower, reduced to ~40% (Figure 6B, Table S19). This is the same condition in which *wDah* laid more eggs than anticipated (Figure 5B), meaning that while *rDah* flies produced the same absolute number of viable eggs in each food condition (Figure 6C, Table S20), *wDah* flies produced significantly fewer viable eggs when maintained under Zn‐limiting conditions (0_100 and 50_50) (Figure 6D, Table S20). Thus, although *wDah* mothers appear to benefit from the same regulatory mechanisms that protect maternal lifespan during dietary Zn restriction, the *white* gene appears to play an important role in their quality control system that maintains high‐quality egg production when dietary Zn levels fall.

## 3 Discussion

Organisms require varying quantities and proportions of nutrients to grow, reproduce, and survive. The macronutrients are well characterised for playing a key role in determining fitness, especially for females who bear the larger anabolic load for offspring production (Lee et al. 2008; Maklakov et al. 2008; Boggs 2009; Simpson and Raubenheimer 2012). The micronutrients, which include vitamins and metal ions, are also critical for these processes, but their effects on reproduction and maternal physiology are less well characterised. Here, we explore the requirement for individual metal ions in *Drosophila* females and find that female flies employ different strategies in response to encountering food that is made without the addition of each of the different metal ions. Of these different responses, we focused on dietary Zn restriction because it elicited a unique combination of phenotypes, causing arrested egg production while preserving maternal lifespan. These data are consistent with dietary Zn being an important indicator of environmental quality that determines resource allocation between life history strategies that shape evolutionary fitness.

Life History Theory proposes that organisms partition limiting resources between reproduction and lifespan to optimise evolutionary fitness (Holliday 1989; Kirkwood and Austad 2000; Flatt and Partridge 2018). Initially, these “resources” were assumed to be derived from the total amount of food, or energy, an organism eats, but more recently, it has become apparent that the relative abundance of specific nutrients in the diet determines the extent to which traits are expressed, independently of how much energy is in the diet (Simpson and Raubenheimer 2012). In particular, the protein:carbohydrate content of the diet has been repeatedly shown to be an important determinant of the inverse relationship between lifespan and reproduction (Lee et al. 2008; Maklakov et al. 2008; Skorupa et al. 2008; Piper and Partridge 2011; Fanson and Taylor 2012; Solon‐Biet et al. 2014). Recently, we have characterised the role of a micronutrient in shaping these traits by studying how varying dietary sterols affect *Drosophila* females (Zanco et al. 2021, 2023). Interestingly, even when mothers consume insufficient sterols to support sustained reproduction, they continue to take cues from dietary protein:carbohydrate proportions to determine the number of eggs they produce. Thus, to lay viable eggs when egg production is high, mothers must commit their own sterol reserves to those eggs, even if it shortens their lifespan. This finding is important because it shows that an essential dietary micronutrient could mediate the way that the macronutrients alter life histories.

Here, our data show that another essential micronutrient, Zn, also plays a key role in determining the way that egg production and lifespan are prioritised—Zn limitation causes rapid cessation of egg production, while maternal lifespan is preserved, or even extended (Kosakamoto et al. 2024). Thus, stopping egg production in response to severe Zn limitation appears to be a strategy for adult flies to maintain adequate levels of body Zn to sustain survival. This inverse relationship between lifespan and reproduction is consistent with the Disposable Soma theory and is similar to what is observed for flies when the dietary protein to carbohydrate ratio is lowered under sterol‐limiting conditions. Together, these data show that while resource‐based lifespan/reproduction trade‐offs can be avoided under some dietary conditions (Grandison, Piper, and Partridge 2009; Piper et al. 2017; Zanco et al. 2021), they can also be revealed in other conditions that differ only in their micronutrient content. Future experiments to determine the interactive effects of varying sterols, protein, carbohydrates, and Zn on life histories will be important to indicate the likely mechanisms by which these priority nutrients alter lifespan.

On a technical note, our data, and that of another study conducted at the same time as this work, reveal information about the range of dietary Zn concentrations over which fly life histories respond (Kosakamoto et al. 2024). At the lower concentrations, recent work from the Obata lab demonstrated that full adult lifespan in flies requires a minimal dietary Zn concentration somewhere between 15 and 1500 ng/L—the level of Zn found as contaminants in their purified agarose and agar, respectively (Kosakamoto et al. 2024). Although the agar we used in our study was not used in theirs, we found that making our food with no added Zn could support full adult lifespan. Thus, our agar is likely to contribute somewhere between 15 and 1500 ng/L Zn. By contrast, we found that egg laying becomes compromised when dietary Zn levels fall below half the concentration used in our reference diet, which corresponds to 12.5 mg/L Zn or less. Thus, *Drosophila* females can sustain full adult lifespan at levels of dietary Zn some 8000 times lower than that required to support full egg production. Interestingly, our data also revealed that there was no benefit or cost to egg laying or lifespan when dietary Zn levels reached twice that required for maximal egg production (25 mg/L; ~16,000 times the minimal level for lifespan). This shows that flies can tolerate a broad range of environmental Zn fluctuations, perhaps emphasising the importance of their evolved ability to store Zn (Fischer, Dieckmann, and Taborsky 2011).

During oogenesis, Zn is actively transported from the adult fly into *Drosophila* oocytes, which is important for egg development and viability (Hu et al. 2020). Given the importance of Zn in oogenesis, we were surprised to find that even after 2 weeks of Zn restriction, females could not adjust their behaviour and/or physiology to maximise egg output when given the choice of food with Zn and food with no Zn added. This is not because the flies lack molecular sensors for Zn: they detect its loss and adjust their physiology to survive. Instead, it appears that their Zn sensing mechanism does not inform the flies' feeding behaviour when the only difference between foods is Zn levels. Interestingly, in another context, severe Zn restriction can increase the flies' preference to feed on yeast over sugar (Kosakamoto et al. 2024). At first glance, this appears to reflect an adaptation to guide the flies to a source of Zn (yeast). But in further assays, the authors found that Zn restriction appears to increase the flies' preference for amino acids, indicating their yeast‐seeking behaviour is due to a change in protein appetite (Ribeiro and Dickson 2010; Vargas et al. 2010; Leitão‐Gonçalves et al. 2017). Further careful dissection of the behavioural changes triggered by Zn restriction will be required to understand how it alters food choice preferences.

We found the flies' inability to maximise egg production in our Zn choice assay especially surprising because we also found that they expressed a preference to lay their eggs on Zn‐containing food. This is consistent with a strategy to ensure offspring survival, as Zn is strictly required for development (Consuegra et al. 2020). *Drosophila* are known to exhibit nutrient‐specific preferences that influence both feeding behaviour and oviposition together (Edgecomb, Harth, and Schneiderman 1994; Lin, Senapati, and Tsao 2019), but this scenario in which a nutrient restriction impacts egg‐laying substrate preference without altering food choice suggests these behaviours are governed by distinct mechanisms. A recent study (Zhu 2015) investigated the neural mechanisms underlying egg‐laying site selection in *Drosophila* and provided evidence that distinct sensory pathways and circuits may be responsible for appetite and oviposition preference. Although they did not study Zn specifically, these findings provide a mechanistic basis for how Zn‐dependent oviposition site preference and food choice might vary. Future work to determine the neural circuits mediating these behaviours should reveal how this has been encoded.

One of the mechanistic findings in our data is that the *white* gene plays an important role in the way that dietary Zn restriction impacts egg viability. *white* encodes a member of the ATP‐binding cassette (ABC) transporter family, known for their role in transporting a wide array of substrates across cellular membranes (Mackenzie et al. 1999; Dean, Rzhetsky, and Allikmets 2001). Recent studies have highlighted the role of *white* in the Malpighian tubules, the fly kidneys, which is key for sequestering excess Zn ions, thus enabling Zn storage in the form of granules that can be retrieved for essential cellular processes as well as acting as a reservoir to prevent cytotoxicity if Zn is in excess (Mackenzie et al. 1999; Yin, Qin, and Zhou 2017; Tejeda‐Guzmán et al. 2018). Mutants in the *white* gene exhibit reduced levels of stored Zn (Tejeda‐Guzmán et al. 2018). The specific mechanism by which the *white* gene influences this is not fully understood, but given its role in transmembrane transport and its expression in the Malpighian tubules (Yin, Qin, and Zhou 2017), it may be involved in the active transport of Zn ions or Zn‐bound complexes across the cellular membranes of the Malpighian tubules. The strongest phenotype we observed in our *white* mutated flies was a large reduction in the viability of eggs, but not their number, when dietary Zn was diluted to 50% of the level in our nutritionally complete food. This suggests that reduced Zn stores may lead to suboptimal Zn levels in the ovaries, which affects egg quality and viability, but not their production. To explore this hypothesis, conducting tissue‐specific RNAi to knock down *white* expression selectively in the Malpighian tubules and measuring its effects on egg viability and Zn levels will be important.

Zn levels are particularly high in the *Drosophila* oocyte; a notable surge in Zn concentration acts as a signal for initiating oocyte maturation, which is crucial for successful fertilisation and embryo growth (Hu et al. 2020). When we restricted dietary Zn, or the flies were fed a diet containing the Zn‐specific chelator TPEN, female egg production was reduced, presumably to avoid producing and laying inviable eggs (Hu et al. 2020). Similar disruptions in oogenesis and decreased fertility have been observed in *C. elegans* hermaphrodites under Zn‐restricted conditions (Hester, Hanna‐Rose, and Diaz 2017), and in humans, Zn deficiency has been linked to a range of adverse reproductive outcomes, including increased risks of infertility, miscarriage, and preterm delivery (Shah and Sachdev 2001; Nossier et al. 2015). Zn deficiency is thought to be prevalent in almost all low‐ and middle‐income countries, in part due to low environmental levels that give rise to Zn‐poor crops (Gupta, Brazier, and Lowe 2020). Thus, Zn limitation, and its effects on reproduction, may be a broadly relevant dietary selection pressure that flies have evolved to monitor and buffer against to optimise their reproductive strategies.

## 4 Conclusion

Our study demonstrates how dietary metal ions can affect both lifespan and reproduction in *Drosophila* females. In particular, we showed that the availability of dietary Zn determines the allocation of resources between reproduction and somatic maintenance, such that lifespan is preserved, even when dietary Zn availability is insufficient. Finally, we found an important role for the *white* gene, an eye pigment transporter, in controlling the production of viable eggs during Zn limitation. These data highlight the importance of metal ions in determining fly life histories and indicate that flies have evolved to include dietary Zn levels in their strategies to maximise reproductive success. Since dietary Zn levels affect reproduction in other organisms, including humans, these data could be useful in providing a platform for understanding how Zn determines whole organism health more broadly.

## 5 Methods

### 5.1 Fly Husbandry

All experiments were conducted using two outbred “wild‐type” *Drosophila* strains called Dahomey (abbreviated here as *rDah*) and white Dahomey (*wDah*). These strains have the same genetic background, but the latter is a homozygous mutant for the *white* gene, which causes the flies to have white eyes (Bingham 1980; Hazelrigg, Levis, and Rubin 1984; Mair, Piper, and Partridge 2005). White‐eyed flies mutant for this gene are commonly used as the genetic background for transgenesis since they provide an easy‐to‐visualise selectable marker to distinguish between transgenics that carry a construct to complement the mutation (orange to red‐eyed) and controls (white‐eyed). *wDah* and *rDah* stocks are maintained in large numbers in continuous overlapping generations in a high‐density population cage at a constant temperature of 25°C, under 12‐h light:dark photoperiods. Upon removal from the population cages, flies were reared for two generations at a controlled density using the eggs laid by age‐matched mothers before use in experiments to control for possible parental effects (Linford et al. 2013). Following the eclosion of the third generation, newly emerged adults were allowed to mate for 48 h before they were lightly anaesthetised with CO_2_ and sorted by sex. All stocks were maintained on sugar yeast food (Bass et al. 2007).

### 5.2 Experimental Diets

The completely defined, synthetic (holidic) diets were prepared using the exome‐matched FLYAA formula as described by Piper et al. (2014) and Piper (2017) (Table S21). For results shown in Figure 1, four different experimental diets were created by diluting down the mixture of all metal ions to 0%, 10%, and 50% of the level in the complete (control) diet. Diets for Figure 2 were prepared by dropping each metal ion separately (Ca, Cu, Fe, Mg, Mn, and Zn). For Figures 3, 4, 5, 6, Zn was diluted to four different concentrations (0%, 10%, 50% and 100%) of the original stock solution (Tables S21 and S22).

### 5.3 Lifespan Assays

For each experimental diet, 10 female flies were placed into each of 10 vials per genotype. Every 2–3 days, flies were transferred into new vials containing fresh food, at which point deaths and censors were recorded (Piper and Partridge 2016) and saved using the software Dlife (Linford et al. 2013). 10 replicates of 10 flies per vial were used per treatment diet.

### 5.4 Fecundity Assays

For the fecundity assay, digital images of the surface of the food with eggs were acquired using a web camera mounted on a Zeiss dissecting microscope, and eggs were counted manually from the images. Egg production was recorded on days 8 and 15 of the experiment after the flies had been exposed to the diets for 24 h. Fecundity was measured as the number of eggs laid per female during each laying period. 10 replicates of 10 flies per vial were used per treatment diet.

### 5.5 Quantification of Metal Ions

To assess the impact of Zn deprivation on metal ion levels, we separated ovaries from the remaining body tissue of *rDah* and *wDah* flies after 8 days of feeding on Zn‐deficient and complete (100N) diets. On the 8th day of adulthood, the flies were anaesthetised using CO_2_, and the ovaries were carefully dissected from the remaining body tissue. Both ovaries and body tissues were collected separately for each genotype. The collected samples were freeze‐dried for a few days until completely dry. Each dried sample was then treated with 50 μL of 65% HNO_3_ and left overnight at room temperature. The samples were subsequently heated at 90°C for 20 min, followed by the addition of 50 μL of H_2_O_2_ and further heating at 70°C for 15 min. The digested samples were diluted to a final volume of 1 mL with deionised water. Metal ion levels (Zn, Cu, Fe, Mg, and Mn) were measured using an Agilent 8800 Triple Quad Inductively Coupled Plasma‐Mass Spectrometer (ICP MS). Calibration was performed using standard solutions for each metal (Figure 4). Five replicates of ovary and body tissues were used for the two treatment diets.

### 5.6 Zinc Choice Assay

Flies for the Zn choice assay were generated in the same way as for the lifespan assay. Treatment conditions consisted of two *Drosophila* maintenance vials, each containing food at their base, with their openings taped together with electrical tape so that flies could freely walk between the two ends to choose their food (Figure S4). The food pairs were a choice condition containing 0% Zn at one end of the vial pair and 100% Zn at the other (0_100); a positive control with 100% Zn food (complete diet) (100_100) at both ends; a negative control with 0% Zn food at both ends (0_0); and a control for random sampling with 50% Zn at both ends (50_50). To control for side preferences unrelated to food composition, vial orientation was noted for each vial pair. Five flies per genotype were placed into each of the 10 connected vial pairs for each treatment condition. Egg number was counted in each vial on days 2, 8 and 15 of adulthood after a 24‐h egg‐laying period, and the side on which each egg was laid was noted.

### 5.7 Egg‐to‐Adult Viability Assay

Egg‐to‐adult viability from each of the choice assay groups was assessed by transferring 10 randomly selected eggs from the diet choice vials to vials containing SY food, which is optimal for fly development. The total number of adults that emerged was counted across the replicate vials for each of the treatments and divided by the number of eggs to give the proportion viable. We ensured equal distribution of eggs from both ends of the diet pairs into separate SY vials. In cases where there weren't enough eggs from one end, we combined eggs from both ends of a diet pair into one SY vial.

### 5.8 Statistical Analyses

Statistical analyses were performed using R Version 2023.06 across various experiments. For survival analyses, linear models were employed to analyse median lifespan. The significance of relationships between genotypes and treatments was evaluated using Type II ANOVA from the package car (Fox and Weisberg 2019). Post hoc pairwise comparisons (Bonferroni adjusted) were conducted for genotypes to evaluate median lifespan differences.

Fecundity responses to metals and Zn concentrations were analysed using Linear Mixed‐Effects Models (LMMs) with the lmer function (Bates et al. 2015). These models assessed fixed effects for day, dilution level, genotype, and their interactions, alongside random effects for replicate variability. Fecundity responses to individual metal ions were analysed using a zero‐inflated Poisson model with day, treatment, genotype, and their interactions as predictors. The glmmTMB function was used to fit the model, specifying a Poisson distribution for the conditional model and a constant zero‐inflation probability. Type III ANOVA (Fox and Weisberg 2019) was used to assess the significance of fixed effects and interactions. Estimated marginal means (EMMs) were calculated using the emmeans package (Lenth et al. 2021) for each treatment within each day and genotype combination. Pairwise comparisons among treatments were performed, specifying a desired order for the comparisons. Compact letter displays (CLDs) were generated using the cld function from the multcomp package (Westfall, Bretz, and Hothorn 2010) to indicate significant differences among treatments, with treatments sharing a letter not being significantly different from each other.

Egg‐to‐adult viability on metal dropouts was analysed using zero‐inflated Poisson models fitted with the glmmTMB function (Brooks et al. 2017). The models included fixed effects for metal dropout, genotype, and their interaction, with Type II ANOVA (Fox and Weisberg 2019) assessing the significance of these effects.

ICP MS analysis of metal concentrations employed linear models with Proportion as the response variable and metal, genotype, and tissue as predictors, including their interactions. Type III ANOVA (Fox and Weisberg 2019) evaluated the overall impact and interactions of these factors, with post hoc pairwise comparisons adjusted using the Tukey method.

In the Zn selective feeding assay, linear mixed‐effects models were used to analyse the impact of dietary Zn variations on egg production. These models incorporated fixed effects for treatment, genotype, day, and their interactions, alongside random effects and diet side for experimental blocks. The significance of fixed effects was evaluated using Type III ANOVA (Fox and Weisberg 2019), with post hoc pairwise comparisons conducted using the emmeans package (Lenth et al. 2021).

To assess whether flies prefer laying eggs in food that contains Zn over food that does not, we first fit the data from the choice scenario (0_100) with a generalised linear mixed effects model (GLMM) using the glmmTMB function (Brooks et al. 2017) using a binomial distribution. In this model, we used the number of flies in the vial as a covariate, day, genotype, and their interactions as fixed effects, and replicate vial and block as random effects. We tested the model for significant fit using a Type III ANOVA (Fox and Weisberg 2019) and then tested if the distribution of eggs across the two vials was significantly different from no choice (assuming a null mean of 0.5) using post hoc tests from the emmeans package (Lenth et al. 2021). This approach allowed us to determine if females show a preference for laying their eggs in Zn‐containing food and how this preference changes over time and differs between genotypes.

To be sure that our no‐choice scenario was not driven by the fact that females tend to lay their eggs together with other eggs, we then compared the choice scenario (0_100) against three no‐choice controls (0_0, 50_50, and 100_100). For each of the control diets, we randomly assigned one of the two vials from each replicate to the treatment group using a custom‐built script in R (see R scripts in FigShare—DOI: 10.26180/26550244). To do this, we employed a generalised linear mixed‐effects model (GLMM) using the glmmTMB function (Brooks et al. 2017) with a binomial distribution. This model included the total number of flies in a vial as a covariate, day, diet, genotype, and their interactions as fixed effects, and replicate vial and block as random effects. After testing the model for a significant fit using a Type III ANOVA (Fox and Weisberg 2019), we compared the choice treatment (0_100) to the three controls (0_0, 50_50, and 100_100) using trt.vs.ctrl custom contrasts in the emmeans package (Lenth et al. 2021). This approach allowed us to determine if the choice treatment resulted in a different distribution of egg‐laying sites than the three control treatments.

For the analysis of egg‐to‐adult viability across different dietary Zn combinations and genotypes, a generalised linear mixed model (GLMM) using the glmmTMB function (Brooks et al. 2017) was employed. The model was specified with a binomial family and logit link to handle the binary nature of the viability data, which consisted of counts of emerged and non‐emerged eggs. The model included fixed effects for treatment and genotype and random effects. The significance of the fixed effects was evaluated using an ANOVA Type III test via the Anova function (Fox and Weisberg 2019). Post hoc comparisons were conducted using the emmeans function, followed by pairwise comparisons.

For the analysis of viable eggs per average female, a zero‐inflated Poisson model was utilised, implemented using the glmmTMB function (Brooks et al. 2017). The significance of the fixed effects was evaluated using a Type III ANOVA (Fox and Weisberg 2019), and subsequent post hoc analyses were conducted to explore specific differences between treatments and genotypes.

For some of the analyses using generalised linear mixed models (GLMMs) fitted with the glmmTMB package, degrees of freedom are sometimes reported as infinite (df = Inf). This is because defining degrees of freedom precisely in GLMMs is challenging due to the complexity of the models and the use of maximum likelihood estimation. Instead, asymptotic normality is often assumed, which allows for approximate inference similar to that in simpler models. This approach is valid when the sample size is large enough, justifying the use of df = Inf, as it reflects the assumption of a sufficiently large sample size for reliable inference.

Plots were produced using ggplot2 (Wickham 2016).

## Author Contributions

**Sweta Sarmah:** conceptualisation, data curation, formal analysis, investigation, methodology, visualisation, writing – original draft. **Hannah Thi‐Hong Hanh Truong:** data curation, investigation. **Gawain McColl:** data curation, investigation. **Richard Burke:** conceptualisation, supervision, writing – review and editing. **Christen K. Mirth:** conceptualisation, formal analysis, funding acquisition, methodology, supervision and writing – review and editing. **Matthew D. W. Piper:** conceptualisation, funding acquisition, methodology, resources, supervision, and writing – review and editing.

## Conflicts of Interest

The authors declare no conflicts of interest.

## Supporting information


Figures S1–S4

Tables S1–S22

## Data Availability

The data that support the findings of this study are available in Figshare (DOI: 10.26180/26550244).
