# Supplementary material for: Dietary Zinc Limitation Dictates Lifespan and Reproduction Trade‐Offs of Drosophila Mothers
Source: Aging Cell. 2025 Jan 31;24(5):e14498. doi: 10.1111/acel.14498 (PMC12073914; doi:10.1111/acel.14498)
Supplement: Supplementary file 2 — Tables S1–S22 [file ACEL-24-e14498-s001.docx]

Table S1.
Anova (Type II) on linear model to best represent the effects of genotypes and dietary metal dilution diets represented as metal concentration on median lifespan

|  | Sum Sq | Df | *P value* |
| --- | --- | --- | --- |
| Genotype | 357 | 1 | 0.181 |
| Metal Concentration (diet) | 367328 | 3 | <0.001^***^ |
| Genotype: Metal Concentration | 5292 | 3 | <0.001^***^ |

Table S2.

Post-hoc pairwise (Bonferroni adjusted) comparisons of genotypes to evaluate median lifespan differences between 100% metal diet (control) and 50%, 10% and 0% metal diets respectively

Genotype= *rDah*

| Contrast | Estimate | df | *p value* |
| --- | --- | --- | --- |
| 100% - 50% | -0.461 | 714 | 0.9963 |
| 100% - 10% | 43.523 | 714 | <0.001^***^ |
| 100%-0% | 55.353 | 714 | <0.001^***^ |

Genotype= *wDah*

| Contrast | Estimate | df | *p value* |
| --- | --- | --- | --- |
| 100% - 50% | -2.741 | 714 | 0.5575 |
| 100% - 10% | 30.639 | 714 | <0.001^***^ |
| 100% - 0% | 44.688 | 714 | <0.001^***^ |

Table S3.
Anova (type III) of the linear model to best represent the relationship between the days on which eggs (age) were counted, genotypes and dietary metal ion concentration

|  | Chisq | Df | *P value* |
| --- | --- | --- | --- |
| Age | 50.92 | 1 | <0.001^***^ |
| Metal Concentration (diet) | 1418.05 | 3 | <0.001^***^ |
| Genotype | 3.52 | 1 | 0.06 |
| Age: Metal Concentration | 20.73 | 3 | <0.001^***^ |
| Age: Genotype | 6.30 | 1 | <0.05^*^ |
| Metal Concentration: Genotype | 32.49 | 3 | <0.001^***^ |
| Age: Metal Concentration: Genotype | 7.87 | 3 | <0.05^*^ |

Table S4.

Anova (Type II) on linear model to best represent the relationship between genotypes, treatment (metal ion restricted diets) and median lifespan

|  | Sum Sq | Df | *P value* |
| --- | --- | --- | --- |
| Genotype | 21994 | 1 | <0.001^***^ |
| Metal diets | 158452 | 6 | <0.001^***^ |
| Genotype: Metal diets | 8090 | 6 | <0.001^***^ |

Table S5.

Post-hoc pairwise comparisons (Bonferroni adjusted) for genotypes to evaluate median lifespan differences between All metal diet (control) and different diets lacking individual metals

Genotype= *rDah*

| Contrast | Estimate | df | *p value* |
| --- | --- | --- | --- |
|  |  |  |  |
| All metals - (-Cu) | 1.055 | 1275 | 1.0000 |
| All metals - (-Ca) | 5.938 | 1275 | 0.2793 |
| All metals - (-Fe) | 3.934 | 1275 | 1.0000 |
| All metals - (-Mg) | 35.501 | 1275 | <0.001^***^ |
| All metals - (-Zn) | -4.258 | 1275 | 1.0000 |
| All metals - (-Mn) | 5.154 | 1275 | 0.7158 |

Genotype= *wDah*

| Contrast | Estimate | df | *p value* |
| --- | --- | --- | --- |
|  |  |  |  |
| All metals - (-Cu) | 6.256 | 1275 | 0.2841 |
| All metals - (-Ca) | 5.499 | 1275 | 0.5159 |
| All metals - (-Fe) | 9.810 | 1275 | <0.01^**^ |
| All metals - (-Mg) | 26.207 | 1275 | <0.0001^***^ |
| All metals - (-Zn) | -8.513 | 1275 | <.05^*^ |
| All metals - (-Mn) | 2.461 | 1275 | 1.0000 |

Table S6.

Anova (type III) of the linear model to best represent the relationship between the days of adulthood (age) when eggs were counted, genotypes and treatment (different metal ion restricted diets) and their interactions. Higher-order interaction terms were removed in a stepwise fashion when removal did not significantly affect model fit (determined by AIC)

|  | Chisq | Df | *P value* |
| --- | --- | --- | --- |
| Age | 471.196 | 1 | <0.001^***^ |
| Metal diets | 2468.271 | 6 | <0.001^***^ |
| Genotype | 17.559 | 1 | <0.001^***^ |
| Age: Metal diets | 167.327 | 6 | <0.001^***^ |
| Age: Genotype | 9.185 | 1 | <0.01^**^ |

Table S7.

Egg production comparison for genotypes across different metal diets and age, measured using Tukey’s multiple comparisons tests, with statistical significance indicated by case-specific letters for age 8 and 15 separately

Genotype= *rDah*

| Age | Metal diets | Df | emmean | Group |
| --- | --- | --- | --- | --- |
| 8 | All metals | Inf | 3.102 | A |
|  | -Cu | Inf | 2.925 | A B |
|  | -Ca | Inf | 2.806 | B |
|  | -Fe | Inf | 2.867 | B |
|  | -Mg | Inf | -1.664 | C |
|  | -Zn | Inf | 0.570 | D |
|  | -Mn | Inf | 2.953 | A B |
| 15 | All metals | Inf | 2.837 | a |
|  | -Cu | Inf | 2.393 | b |
|  | -Ca | Inf | 2.425 | b |
|  | -Fe | Inf | 2.326 | b |
|  | -Mg | Inf | -2.909 | c |
|  | -Zn | Inf | -0.256 | c |
|  | -Mn | Inf | 2.445 | b |

Genotype= *wDah*

| Age | Metal diets | Df | emmean | Group |
| --- | --- | --- | --- | --- |
| 8 | All metals | Inf | 2.998 | A |
|  | -Cu | Inf | 2.821 | A B |
|  | -Ca | Inf | 2.702 | B |
|  | -Fe | Inf | 2.763 | B |
|  | -Mg | Inf | -1.768 | C |
|  | -Zn | Inf | 0.466 | D |
|  | -Mn | Inf | 2.849 | A B |
| 15 | All metals | Inf | 2.733 | a |
|  | -Cu | Inf | 2.289 | b |
|  | -Ca | Inf | 2.321 | b |
|  | -Fe | Inf | 2.221 | b |
|  | -Mg | Inf | -3.014 | c |
|  | -Zn | Inf | -0.360 | c |
|  | -Mn | Inf | 2.342 | b |

Table S8.

Anova (type II) of the zero-inflated model to best represent the relationship between egg-to-adult viability of eggs collected from different metal diets and genotypes (*rDah* and *wDah*)

|  | Chisq | Df | *P value* |
| --- | --- | --- | --- |
| Metal diets | 5.83 | 4 | 0.2119 |
| Genotype | 1.33 | 1 | 0.2496 |
| Metal dropout: Genotype | 3.57 | 4 | 0.4668 |

Table S9.
Anova (Type II) on linear model to best represent the relationship between genotypes, zinc dilution diets represented as zinc concentration and median lifespan

|  | Sum Sq | Df | *p value* |
| --- | --- | --- | --- |
| Genotype | 2752 | 1 | 0.004^**^ |
| Zinc Concentration (diet) | 1606 | 3 | 0.184 |
| Genotype: Treatment | 1119 | 3 | 0.338 |

Table S10.

Post-hoc pairwise comparisons (Bonferroni adjusted) for genotypes to evaluate lifespan differences between 100% zinc diet (control) and 50%, 10% and 0% zinc diets respectively

Genotype= *rDah*

| Contrast | Estimate | df | *p value* |
| --- | --- | --- | --- |
| 100% - 50% | 2.957 | 678 | 1.0000 |
| 100% - 10% | -2.807 | 678 | 1.0000 |
| 100% - 0% | 0.825 | 678 | 1.0000 |

Genotype= *wDah*

| Contrast | Estimate | df | *p value* |
| --- | --- | --- | --- |
| 100% - 50% | -2.196 | 678 | 1.0000 |
| 100% - 10% | -2.816 | 678 | 1.0000 |
| 100% - 0% | 2.268 | 678 | 1.0000 |

Table S11.
Anova (type III) of the linear model to best represent the relationship between the days of adulthood (age) the eggs counted, genotypes and dietary Zinc concentration

|  | Chisq | Df | *p value* |
| --- | --- | --- | --- |
| Age | 235.34 | 1 | <0.001^***^ |
| Zinc Concentration | 825.10 | 3 | <0.001^***^ |
| Genotype | 24.07 | 1 | <0.001^***^ |
| Age: Zinc Concentration | 56.00 | 3 | <0.001^***^ |
| Age: Genotype | 0.16 | 1 | 0.68 |
| Zinc Concentration: Genotype | 15.65 | 3 | <0.01^**^ |
| Age: Zinc Concentration: Genotype | 16.67 | 3 | <0.001^***^ |

Table S12.

Anova (type III) of the linear model representing the relationship between the proportions of metals measured (where each metal is expressed as a proportion of that flies fed on 0Zn diets, divided by that found in flies feeding on a complete diet), tissues (body & ovary) and genotypes (*rDah* and *wDah*)

|  | Sum Sq | Df | *p value* |
| --- | --- | --- | --- |
| Metal | 16.166 | 4 | <0.001^***^ |
| Genotype | 0.211 | 1 | <0.001^***^ |
| Tissue | 10.566 | 1 | <0.001^***^ |
| Metal: Genotype | 1.596 | 4 | <0.001^***^ |
| Metal: Tissue | 5.688 | 4 | <0.001^***^ |
| Genotype: Tissue | 0.196 | 1 | <0.001^***^ |
| Metal: Genotype: Tissue | 0.522 | 4 | <0.001^***^ |

Table S13.

Differences in metal levels across tissues and genotypes, measured using Tukey’s multiple comparisons tests

| Tissue | Metal | Df | emmean | Group |
| --- | --- | --- | --- | --- |
| *Body* | *rDah* | | | |
|  | Zn | 480 | 0.153 | 1 |
|  | Fe | 480 | 0.917 | 3 |
|  | Mg | 480 | 1.029 | 4 5 6 |
|  | Mn | 480 | 1.069 | 5 6 |
|  | Cu | 480 | 1.126 | 6 |
|  | *wDah* | | | |
|  | Zn | 480 | 0.528 | 2 |
|  | Fe | 480 | 0.993 | 3 4 5 |
|  | Mg | 480 | 1.069 | 5 6 |
|  | Mn | 480 | 0.954 | 3 4 |
|  | Cu | 480 | 1.256 | 7 |
| *Ovary* | *rDah* | | | |
|  | Zn | 480 | 0.362 | 1 |
|  | Fe | 480 | 0.223 | 4 |
|  | Mg | 480 | 0.203 | 3 4 |
|  | Mn | 480 | 0.145 | 2 3 4 |
|  | Cu | 480 | 1.126 | 3 4 |
|  | *wDah* | | | |
|  | Zn | 480 | 0.101 | 1 2 |
|  | Fe | 480 | 0.132 | 1 2 3 4 |
|  | Mg | 480 | 0.156 | 2 3 4 |
|  | Mn | 480 | 0.112 | 1 2 3 |
|  | Cu | 480 | 1.256 | 2 3 4 |

Table S14.

Anova (type III) of the linear mixed model to best represent the relationship between egg production, treatment (diet combinations with dietary Zn variations), days of adulthood (age) and genotype (*rDah* or *wDah)*

|  | Chisq | Df | *p value* |
| --- | --- | --- | --- |
| Treatment (diet) | 196.90 | 3 | <0.001^***^ |
| Genotype | 17.12 | 1 | <0.001^***^ |
| Age | 1221.46 | 2 | <0.001^***^ |
| Treatment: Genotype | 10.32 | 3 | 0.016^*^ |
| Treatment: Age | 81.02 | 6 | <0.001^***^ |
| Genotype: Age | 40.29 | 2 | <0.001^***^ |
| Treatment: Genotype: Age | 6.50 | 6 | 0.369 |

Table S15.

Differences between egg production on different treatments (diet combinations with dietary Zn variations) counted on different days of adulthood (age) and genotypes measured using Tukey’s multiple comparisons tests

| Age | Treatment (diet) | emmean | Df | Group |
| --- | --- | --- | --- | --- |
| 2 | *rDah* | | | |
|  | 0_100 | 32.657 | 8.92 | 1 |
|  | 0_0 | 32.838 | 25.47 | 1 |
|  | 50_50 | 34.692 | 25.47 | 12 |
|  | 100_100 | 39.492 | 25.47 | 2 |
|  | *wDah* | | | |
|  | 0_100 | 27.560 | 8.92 | 1 |
|  | 0_0 | 24.598 | 25.47 | 1 |
|  | 50_50 | 27.998 | 25.47 | 1 |
|  | 100_100 | 29.548 | 25.47 | 1 |
| 8 | *rDah* | | | |
|  | 0_100 | 17.503 | 8.92 | 2 |
|  | 0_0 | 2.968 | 25.47 | 1 |
|  | 50_50 | 19.959 | 25.47 | 2 |
|  | 100_100 | 28.128 | 25.47 | 3 |
|  | *wDah* | | | |
|  | 0_100 | 17.503 | 8.92 | 2 |
|  | 0_0 | 2.968 | 25.47 | 1 |
|  | 50_50 | 19.959 | 25.47 | 3 |
|  | 100_100 | 28.128 | 25.47 | 23 |
| 15 | *rDah* | | | |
|  | 0_100 | 6.683 | 8.92 | 2 |
|  | 0_0 | 0.615 | 25.47 | 1 |
|  | 50_50 | 6.065 | 25.47 | 12 |
|  | 100_100 | 9.278 | 25.47 | 2 |
|  | *wDah* | | | |
|  | 0_100 | 8.867 | 8.92 | 2 |
|  | 0_0 | 1.192 | 25.47 | 1 |
|  | 50_50 | 9.288 | 25.47 | 2 |
|  | 100_100 | 10.105 | 25.47 | 2 |

Table S16.

Analysis of Deviance (Type III Wald Chisquare Tests) of the generalized linear mixed model to represent the relationship between egg-laying preferences and factors including total number of flies, treatment conditions (diet combinations with dietary Zn variations), days of adulthood (Age), and genotypes (*rDah* or *wDah*)

|  | Chisq | Df | *p value* |
| --- | --- | --- | --- |
| Total flies | 5.34 | 1 | <0.05^*^ |
| Treatment (diet) | 4.45 | 3 | 0.217 |
| Age | 13.61 | 2 | <0.01^**^ |
| Genotype | 25.59 | 1 | <0.001^***^ |
| Treatment: Day | 214.19 | 6 | <0.001^***^ |
| Treatment: Genotype | 122.07 | 3 | <0.001^***^ |
| Age: Genotype | 1.59 | 2 | 0.451 |
| Treatment: Day: Genotype | 83.66 | 6 | <0.001^***^ |

Table S17.

Comparing egg-laying preferences across diet combinations with dietary Zn Variations, days of adulthood (Age), and genotypes (*rDah* or *wDah*) using Tukey's multiple comparisons tests

| Age | Contrast | Estimate | df | *p value* |
| --- | --- | --- | --- | --- |
| 2 | *rDah* | | | |
|  | (0_0)- (0_100) | -0.057 | Inf | 0.976 |
|  | (100_100)- (0_100) | -0.461 | Inf | 0.115 |
|  | (50_50)- (0_100) | -0.038 | Inf | 0.989 |
|  | *wDah* | | | |
|  | (0_0)- (0_100) | -0.042 | Inf | 0.9874 |
|  | (100_100)- (0_100) | -0.969 | Inf | <0.001^***^ |
|  | (50_50)- (0_100) | 0.429 | Inf | 0.164 |
| 8 | *rDah* | | | |
|  | (0_0)- (0_100) | -0.707 | Inf | <0.05^*^ |
|  | (100_100)- (0_100) | -0.239 | Inf | 0.598 |
|  | (50_50)- (0_100) | -0.894 | Inf | <0.001^***^ |
|  | *wDah* | | | |
|  | (0_0)- (0_100) | -0.579 | Inf | 0.102 |
|  | (100_100)- (0_100) | -1.453 | Inf | <0.001^***^ |
|  | (50_50)- (0_100) | -1.501 | Inf | <0.001^***^ |
| 15 | *rDah* | | | |
|  | (0_0)- (0_100) | -1.497 | Inf | <0.001^***^ |
|  | (100_100)- (0_100) | -0.594 | Inf | <0.05^*^ |
|  | (50_50)- (0_100) | -1.179 | Inf | <0.001^***^ |
|  | *wDah* | | | |
|  | (0_0)- (0_100) | -1.767 | Inf | <0.001^***^ |
|  | (100_100)- (0_100) | -1.094 | Inf | <0.001^***^ |
|  | (50_50)- (0_100) | -1.067 | Inf | <0.001^***^ |

Table S18.

Anova (type III) of the zero-inflated model to best represent the relationship between egg-to-adult viability of eggs collected from different treatments (diet combinations with dietary Zn variations), and genotypes (*rDah* or *wDah*)

|  | Chisq | Df | *p value* |
| --- | --- | --- | --- |
| Treatment (diet) | 5.06 | 2 | 0.079 |
| Genotype | 0.01 | 1 | 0.903 |
| Treatment: Genotype | 17.14 | 2 | <0.001^***^ |

Table S19.

Differences in the proportion of eggs that gave rise to viable adults across different treatments (diet combinations with dietary zinc variations). Data analyses were performed on genotypes separately using Tukey’s multiple comparisons tests

Genotype= *rDah*

| Treatment (diet) | emmean | Group |
| --- | --- | --- |
| 100_100 | 4.655 | 1 |
| 0_100 | 3.017 | 1 |
| 50_50 | 2.154 | 1 |

Genotype= *wDah*

| Treatment (diet) | emmean | Group |
| --- | --- | --- |
| 100_100 | 4.783 | 2 |
| 0_100 | 2.960 | 2 |
| 50_50 | -0.485 | 1 |

Table S20.

Differences in the absolute number of viability eggs across different treatments (diet combinations with dietary zinc variations), and genotypes measured using Tukey’s multiple comparisons tests

Genotype= *rDah*

| Treatment (diet) | emmean | Group |
| --- | --- | --- |
| 100_100 | 2.77 | 1 |
| 0_100 | 2.56 | 1 |
| 50_50 | 2.58 | 1 |

Genotype= *wDah*

| Treatment (diet) | emmean | Group |
| --- | --- | --- |
| 100_100 | 2.76 | 3 |
| 0_100 | 2.38 | 2 |
| 50_50 | 1.40 | 1 |

Table S21.

FLYAA (Complete holidic diet- also referred to as 100N)

| Nutrients | Ingredients | Stock | Amount/L | Supplier (order code) |
| --- | --- | --- | --- | --- |
| MilliQ Water |  |  | 777 ml |  |
| Gelling Agent | Agar |  | 7.00 g | Sigma Aldrich (A7002) |
| Amino Acids | L-isoleucine |  | 0.56 g | Sigma Aldrich (I2752) |
|  | L-leucine |  | 1.02 g | Sigma Aldrich (L8912) |
|  | L-tyrosine |  | 0.46 g | Sigma Aldrich (T8566) |
| Carbohydrates | Sucrose |  | 17.12 g | Sigma Aldrich (S1888) |
| Sterols | Cholesterol | 20 mg/ml in EtOH | 15 ml | Glentham Life Sciences (GE0100) |
| Metal ions | CaCl_2_.2H_2_O | 1000x: 250 g/l | 1 ml | Sigma Aldrich (C7902) |
|  | CuSO_4_.5H_2_O | 1000x: 2.5g/l | 1 ml | Sigma Aldrich (C7631) |
|  | FeSO_4_.7H_2_O | 1000x: 25 g/l | 1 ml | Sigma Aldrich (F7002) |
|  | MgSO4 (anhydrous) | 1000x: 250 g/l | 1 ml | Sigma Aldrich (M7506) |
|  | MnCl_2_.4H_2_O | 1000x: 1 g/l | 1 ml | Sigma Aldrich (M3634) |
|  | Zn SO_4_.7H_2_O | 1000x: 25 g/l | 1 ml | Sigma Aldrich (Z0251) |
| Base | Buffer | 10x:  30ml/l glacial acetic acid | 100 ml | Merck (100063) |
|  |  | 30g/l KH_2_PO_4_ |  | Sigma Aldrich (P9791) |
|  |  | 10g/l NaHCO_3_ |  | Sigma Aldrich (S8875) |
| Essential Amino Acids (EAA) | L-arginine HCL | 8 g/l | 30.2 ml | Sigma Aldrich (A5131) |
|  | L-histidine | 10g/l |  | Sigma Aldrich (H8000) |
|  | L-lysine | 19 g/l (HCL) |  | Sigma Aldrich (L5626) |
|  | L-methionine | 8 g/l |  | Sigma Aldrich (M9625) |
|  | L-phenylalanine | 13 g/l |  | Sigma Aldrich (P2126) |
|  | L-threonine | 20 g/l |  | Sigma Aldrich (T8625) |
|  | L-tryptophan | 5 g/l |  | Sigma Aldrich (T0254) |
|  | L-valine | 28 g/l |  | Sigma Aldrich (V0500) |
| Non-Essential Amino Acids (NEAA) | L-alanine | 35 g/l | 30.2 ml | Sigma Aldrich (A7627) |
|  | L-asparagine | 17 g/l |  | Sigma Aldrich (A0884) |
|  | L-aspartic acid | 17 g/l |  | Sigma Aldrich (A6683) |
|  | L-glutamine | 25 g/l |  | Sigma Aldrich (G5889) |
|  | Glycine | 32 g/l |  | Sigma Aldrich (G7126) |
|  | L-proline | 15 g/l |  | Sigma Aldrich (P0380) |
|  | L-serine | 19 g/l |  | Sigma Aldrich (S4500) |
|  | L-glutamic acid | 100 g/l | 7.59 ml | Sigma Aldrich (G5889) |
|  | L-cysteine | 1 g/l | 3.41 ml | SigmaAldrich (C7477) |
| Vitamins | Thiamine | 47.6x: 0.067g/l | 21 ml | Sigma Aldrich (T4625) |
|  | Riboflavin | 47.6x: 0.033g/l |  | Sigma Aldrich (R4500) |
|  | Nicotinic Acid | 47.6x: 0.399g/l |  | Sigma Aldrich (N4126) |
|  | Ca pantothenate | 47.6x: 0.516g/l |  | Sigma Aldrich (21210) |
|  | Pyridoxine | 47.6x: 0.083g/l |  | Sigma Aldrich (P9755) |
|  | Biotin | 47.6x: 0.007g/l |  | Sigma Aldrich (B4501) |
| Folate | Folic Acid | 1000x: 0.5g/l | 1 ml | Sigma Aldrich (F7876) |
| Other nutrients | Choline Chloride | 125x: 6.25g/l | 8 ml | Sigma Aldrich (C1879) |
|  | Myo-inositol | 125x: 0.63g/l |  | Sigma Aldrich (I7508) |
|  | Inosine | 125x: 8.13g/l |  | Sigma Aldrich (I4125) |
|  | Uridine | 125x: 7.50g/l |  | Sigma Aldrich (U3750) |
| Preservatives | Nipagin | 100 g/l methyl 4-hydroxybenzoate in 95% EtOH | 15 ml | Sigma Aldrich (W271004) |
|  | Propionic Acid |  | 6 ml | Merck (8.00605) |

Table S22.

Modified holidic diets (FLYAA)

| Diet | Modifications |
| --- | --- |
| Calcium restricted (-Ca) | Diet made without adding calcium |
| Magnesium restricted (-Mg) | Diet made without adding magnesium |
| Copper restricted (-Cu) | Diet made without adding copper |
| Iron restricted (-Fe) | Diet made without adding Iron |
| Manganese restricted (-Mn) | Diet made without adding Manganese |
| Zinc restricted (-Zn) | Diet made without adding Zinc |
| 100% metals/ 100% Zn/ All metals/ 100N | Complete diet. No manipulations |
| 50% metals | Each metal added to the diet were diluted to 50% of their original stock solution |
| 10% metals | Each metal added to the diet were diluted to 10% of their original stock solution |
| 0% metals | No metals were added to the diet |
| 50% Zn | The zinc added to the diet was diluted to 50% of its original stock solution |
| 10% Zn | The zinc added to the diet was diluted to 10% of its original stock solution |
| 0% Zn | No zinc was added to the diet |

^^[[1]](#footnote-2)^^

1. For 10% dilution: Add 5ml of stock solution to 45ml of miliQ water to make a 50ml diluted solution.

   For 50% dilution: Add 25ml of stock solution to 25ml of miliQ water to make a 50ml diluted solution.

   For 100% dilution: Substitute the metal ions in the holidic food (e.g., zinc, iron, copper, manganese, and magnesium) with 1ml of miliQ water for each in 1L holidic food.

   For 0% dilution: Follow the 100% recipe without any changes. [↑](#footnote-ref-2)
